# Immunoglobulin A Vasculitis: Contemplating Treatment for Gastrointestinal Involvement

**DOI:** 10.7759/cureus.39405

**Published:** 2023-05-23

**Authors:** Ernesto R Lopez Castillo, Osman Bhatty

**Affiliations:** 1 Internal Medicine, Advocate Illinois Masonic Medical Center, Chicago, USA; 2 Rheumatology, Advocate Illinois Masonic Medical Center, Chicago, USA

**Keywords:** henoch-schönlein purpura, case report, corticosteroids, iga vasculitis histopathology, abdominal pain, rash, iga vasculitis

## Abstract

This is a case of a 30-year-old female with a history of recent cholecystectomy who presented with a chief complaint of diffuse rash, abdominal pain, vomiting, and diarrhea. Infectious and autoimmune tests were unrevealing, but a skin biopsy confirmed the presence of immunoglobulin A (IgA) vasculitis. Worsening gastrointestinal (GI) symptoms prompted the care team to pursue upper and lower endoscopies, which were suggestive of GI involvement of IgA vasculitis. The patient responded well to corticosteroids and later had a recurrence of diarrhea which improved with cholestyramine, raising the question of a co-existent post-cholecystectomy syndrome. This case highlights the importance of having broad differential diagnoses, and establishing the extent of organ involvement in IgA vasculitis, as this can dictate the type of treatment used.

## Introduction

Immunoglobulin A (IgA) vasculitis, formerly known as Henoch-Schönlein purpura, is a multiorgan disease that affects the skin, joints, kidneys, and gastrointestinal (GI) tract through immune complex deposition. Although many triggers have been associated with it (i.e., viral infections, vaccines, drugs), no apparent underlying cause has been identified [1]. It is typically seen in children and is considered to be rare in adults [2]. The annual incidence of IgA vasculitis is estimated at 0.8-1.8/100,000 in adults and 3-26.7/100,000 in children [3].

The classic presentation of IgA vasculitis includes a triad of purpuric rash, arthritis/arthralgia, and abdominal pain, ranging from mild self-limited illness to severe, life-threatening disease [4]. Skin biopsy is the gold standard for diagnosis. Deposits of IgA immune complexes can be appreciated with immunofluorescence, but immunoglobulin G (IgG), immunoglobulin M (IgM), C3, or fibrin can also be seen [5].

Determining if organ dysfunction, particularly GI tract involvement, is from IgA vasculitis can be challenging given the nonspecific clinical findings [2,4]. A careful and thorough workup must be performed to rule out relevant etiologies given the patient’s clinical presentation.

We report the case of an adult female who developed IgA vasculitis with skin and GI tract involvement days after an elective cholecystectomy.

## Case presentation

A 30-year-old female presented to the emergency department with a chief complaint of diffuse rash, vomiting, and diarrhea accompanied by abdominal pain, which started two days after an elective cholecystectomy that was performed two weeks prior. The rash began in her arms and legs and then spread all over her body, including her gallbladder surgery scars. Her medical history included gastroesophageal reflux disease and irritable bowel syndrome with diarrhea. Her family history was unremarkable. She denied drug allergies, recent vaccinations, or upper respiratory tract infections.

**Figure 1:**
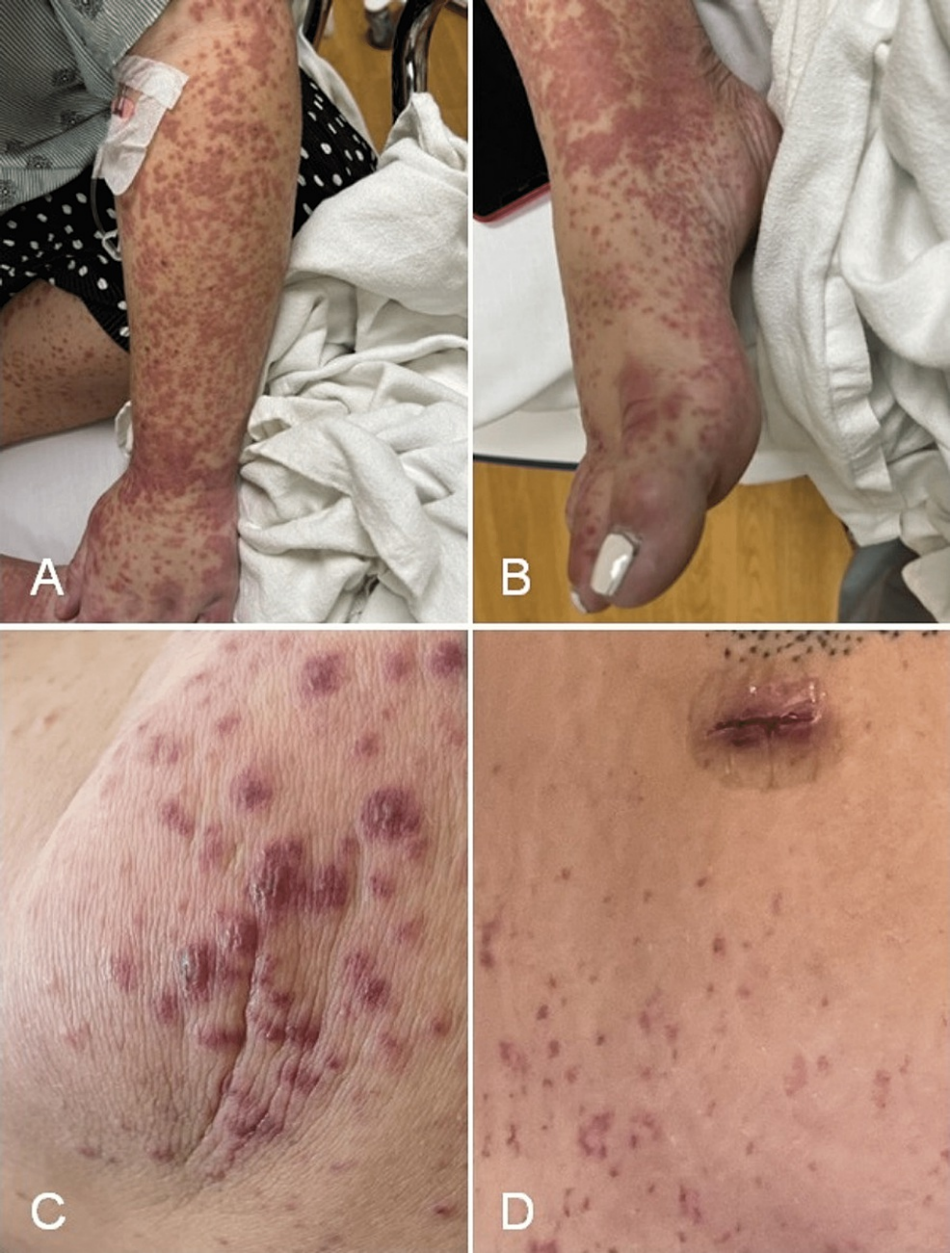
Palpable maculopapular rash located on the left arm (A) and right foot (B). A close-up view of the patient’s right knee with a better appreciation of the rash’s characteristics (C). Cholecystectomy surgical scar with purpuric borders (D).

On physical examination, a palpable, non-blanching, maculopapular, purpuric rash was noted over the face, neck, trunk, and upper and lower extremities, including palms and soles (Figure 1). The epigastrium and left upper quadrant were tender to palpation. No joint swelling was appreciated.

Initial lab work was notable for elevated white blood cell count of 13.8 K/µL (normal range: 4.2-11.0 K/µL), erythrocyte sedimentation rate of 43 mm/hour (normal range: 0-20 mm/hour), and C-reactive protein of 9.9 mg/dL (normal range: <1.0 mg/dL). Platelets and coagulation studies were normal. Kidney function and liver function tests were within normal limits. Urinalysis was unrevealing. The infectious workup was negative.

Immunologic tests including anti-nuclear antibody, complement C3 and C4, total complement activity, anti-neutrophil cytoplasmic antibody (ANCA) IgG, myeloperoxidase antibody, proteinase-3 antibody, tissue transglutaminase antibody IgG and IgA, and cryoglobulins were unremarkable. Extensive infectious workup including blood cultures, gastrointestinal pathogen panel, stool culture, *Clostridium difficile* toxin, viral hepatitis panel, human immunodeficiency virus, rapid plasma reagin, and severe acute respiratory syndrome coronavirus 2 yielded negative results.

**Figure 2:**
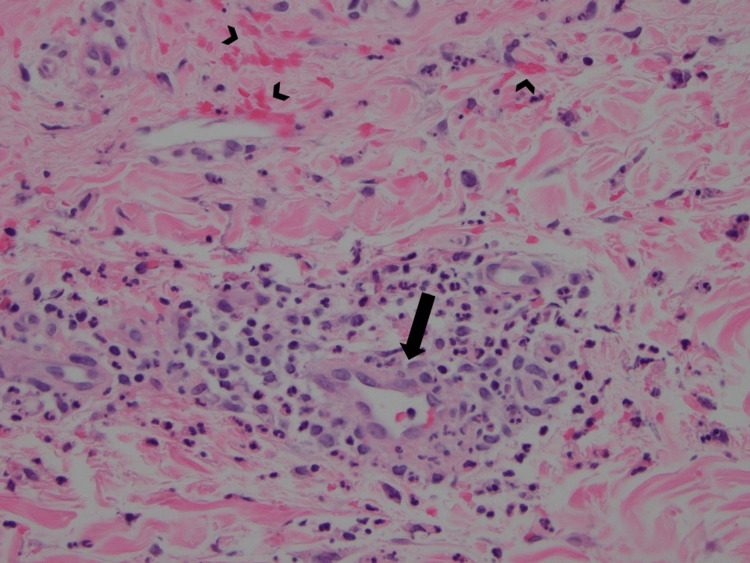
Skin biopsy specimen with hematoxylin and eosin staining showing superficial perivascular inflammation with infiltration of the vessel wall with neutrophils (arrow), along with karyorrhexis and red blood cell extravasation (arrowheads), consistent with leukocytoclastic vasculitis.

Abdominal computed tomography (CT) showed mild circumferential wall thickening of the proximal small bowel with inflammatory mesenteric fat stranding. A full-thickness punch biopsy of the skin revealed leukocytoclastic vasculitis (Figure 2) with IgA and C3 deposition consistent with IgA vasculitis.

**Figure 3:**
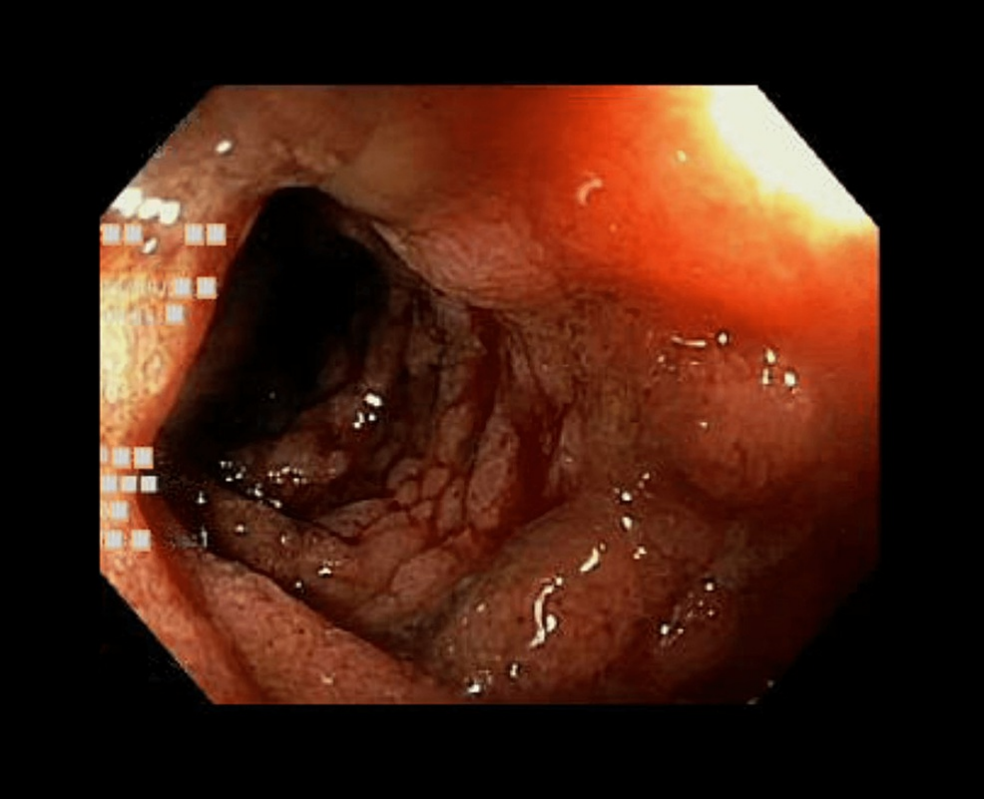
Colonoscopy image of the terminal ileum showing the edematous mucosa with the presence of blood.

**Figure 4:**
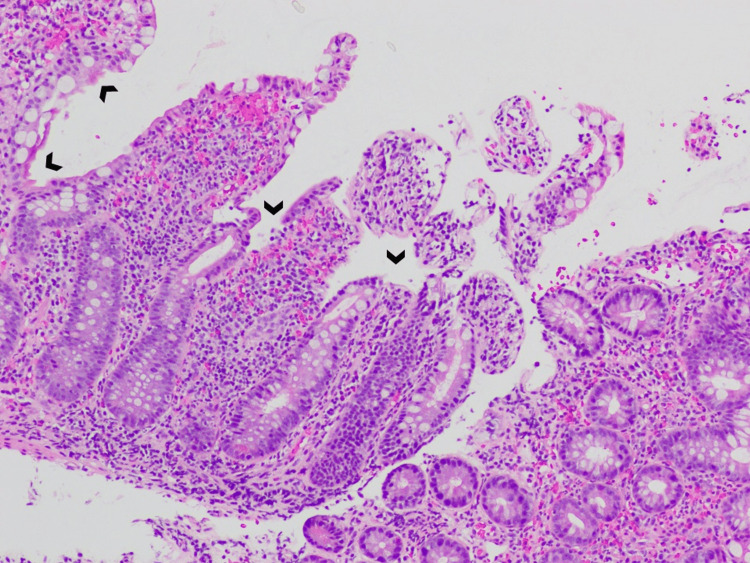
Ileal mucosa with erosions (arrowheads).

**Figure 5:**
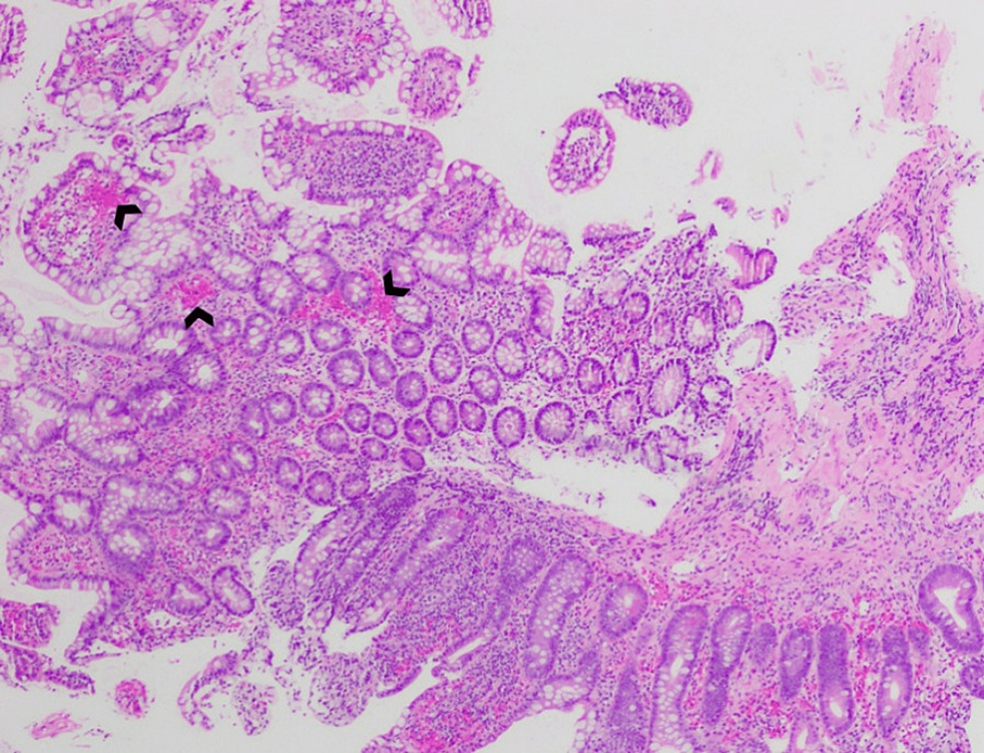
Ileal mucosa with expanded lamina propria and multiple areas of hemorrhage (arrowheads).

**Figure 6:**
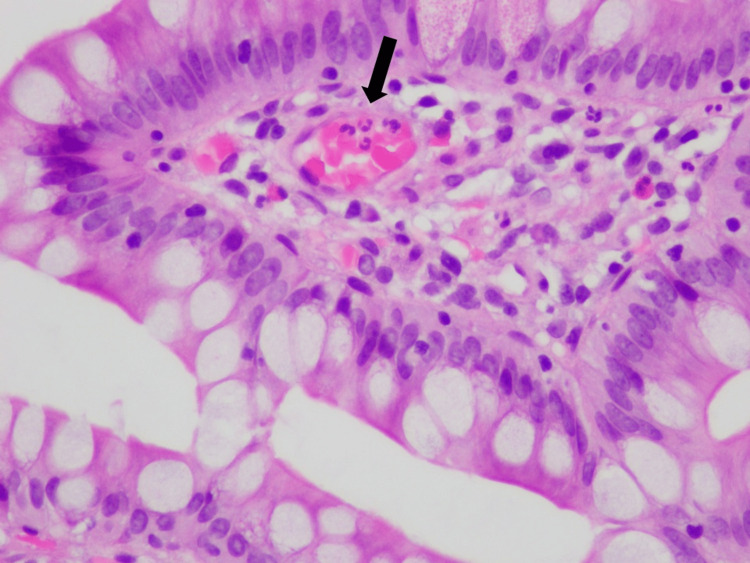
Ileal mucosa displaying neutrophils associated with capillary walls without definite endothelial damage, a finding consistent with capillaritis (arrow).

By her third day of admission, the patient had developed poor oral intake, persistent vomiting, diarrhea, and new-onset hematochezia. Methylprednisolone 60 mg intravenous for suspected GI involvement of IgA vasculitis was initiated, and GI endoscopy was recommended. Diffuse mildly erythematous mucosa with no stigmata of bleeding was found in the duodenal bulb in the first and second portions of the duodenum. In the entire colon and terminal ileum, mild inflammation characterized by adherent blood, edema, erosions, erythema, friability, granularity, and mucus was seen (Figure 3). Colonic and rectal biopsies were negative, but biopsy from the small intestine was notable for peptic duodenitis, active ileitis with erosions (Figure 4), lamina propria hemorrhage (Figure 5), and capillaritis (Figure 6). Considering the unremarkable extensive immunologic and infectious workup, these findings strongly suggested IgA vasculitis as the cause of her symptoms.

The patient experienced a steady improvement and was discharged on a prednisone taper. Five days later, the abdominal pain continued to improve, but diarrhea suddenly recurred, so cholestyramine was started for suspected post-cholecystectomy syndrome. Together this resulted in the complete resolution of her symptoms. At the three-month follow-up, the patient was asymptomatic, with no skin rash, and happy to feel like herself again.

## Discussion

This is a case of an adult female with idiopathic IgA vasculitis presenting with skin and GI manifestations requiring urgent corticosteroid treatment. Some studies comparing children and adults with IgA vasculitis report that adults tend to have less frequency of GI and joint disease [6,7] but more frequent and severe renal disease [6-8].

The rash in IgA vasculitis is usually seen in dependent body areas such as the lower extremities [4]. This case stands out for having a whole-body distribution, even affecting the gallbladder surgery scars. During the initial workup of IgA vasculitis, it is important to rule out other diagnoses that could present with a similar skin rash. A thorough autoimmune panel was performed for our patient, which helped rule out conditions such as systemic lupus erythematosus, ANCA-associated vasculitides, and cryoglobulinemic vasculitis. Ultimately, a skin biopsy is the gold standard for diagnosis of IgA vasculitis, with expert literature suggesting it should be performed within 48 hours of the appearance of the lesion to increase diagnostic yield [5]. Another highlight of this case is that characteristic histopathologic and immunofluorescence findings were seen even though our patient had presented two weeks after the onset of the rash. Regardless, pursuing a skin biopsy as early as possible is encouraged.

GI involvement in IgA vasculitis is not uncommon. A study of 260 adult patients with IgA vasculitis by Audemard-Verger et al. reported that 137 (53%) patients had GI symptoms, of whom abdominal pain was seen in 135 (99%), intestinal bleeding in 43 (31%), and diarrhea in 36 (26%) patients [9]. Such unspecific clinical manifestations often require additional testing modalities. Abdominal CT mainly shows thickening of the intestinal bowel wall [9], while GI endoscopies show an array of lesions, such as ulcerations, mucosal erythema, and purpura [10]. Vasculitis is not commonly found on small bowel biopsy [9]; rather, active duodenitis with erosions and lamina propria hemorrhage are frequently noted [10-12]. Our patient’s histopathologic findings were similar to what is commonly described in the literature, except for the presence of significant ileal disease, which is not frequent [10-12].

Various studies have reported cases of IgA vasculitis with GI involvement where abdominal CT imaging and GI endoscopy are frequently obtained to characterize the extent of the disease, especially when characteristic skin findings or biopsy results are absent. For example, Matsumura et al. reported a case without skin findings where abdominal CT imaging and GI endoscopy were necessary to establish the diagnosis [13]. Other authors have also considered GI endoscopy a critical step before considering immunosuppressive therapy [9,14]. Initially, in our case, after an extensive negative laboratory workup, it was not entirely clear whether the GI symptoms were secondary to a post-cholecystectomy syndrome, bile acid diarrhea, or IgA vasculitis until abdominal CT imaging revealed small bowel wall thickening. Later, when the patient developed hematochezia, pursuing a GI endoscopy was the pivotal step to confirming the diagnosis. It helped rule out other mimickers such as inflammatory bowel disease, infectious colitis, malignancy, diverticulosis, and hemorrhoids.

Treatment of IgA vasculitis with GI involvement is generally conservative, with most patients experiencing a self-limiting course [2,9]. Treatment with corticosteroids may be necessary for severe disease to hasten symptom resolution; however, corticosteroids do not alter long-term outcomes [15]. The duration of therapy remains a matter of debate. In our case, the decision to treat was supported by the patient’s poor oral intake, incapacitating symptoms, and progressive hematochezia; eventually, symptom control was achieved after a few days. The recurrence of diarrhea, which improved with cholestyramine, raised the suspicion for potential co-existence of a post-cholecystectomy syndrome, exemplifying again that a broad differential should be maintained. Additionally, this case shows that close monitoring and follow-up are essential to ensure that patients are appropriately managed and that any complications or disease recurrence are promptly identified and treated.

## Conclusions

When IgA vasculitis is suspected, it is crucial to have a broad differential given that many other diseases may act as mimickers or co-exist with this condition. Imaging studies and endoscopies are essential in establishing the diagnosis and determining the extent of GI involvement, which could dictate the type of treatment used. While corticosteroids may be effective in hastening symptom resolution, close monitoring and follow-up are essential to ensure appropriate disease control.
